# Sperm Transcripts Associated With Odorant Binding and Olfactory Transduction Pathways Are Altered in Breeding Bulls Producing Poor-Quality Semen

**DOI:** 10.3389/fvets.2022.799386

**Published:** 2022-02-22

**Authors:** Thirumalaisamy Karuthadurai, Dayal Nitai Das, Arumugam Kumaresan, Manish Kumar Sinha, Elango Kamaraj, Pradeep Nag, John Peter Ebenezer Samuel King, Tirtha Kumar Datta, Ayyasamy Manimaran, Sakthivel Jeyakumar, Kerekoppa Ramesha

**Affiliations:** ^1^Theriogenology Laboratory, Veterinary Gynaecology and Obstetrics, Southern Regional Station of ICAR-National Dairy Research Institute, Bengaluru, India; ^2^Dairy Production Section, Southern Regional Station of ICAR-National Dairy Research Institute, Bengaluru, India; ^3^Animal Genomics Laboratory, ICAR-National Dairy Research Institute, Karnal, India

**Keywords:** breeding bulls, semen quality, transcriptomics, olfactory pathway, odorant binding

## Abstract

Spermatozoa carries a reservoir of mRNAs regulating sperm functions and fertilizing potential. Although it is well recognized that a considerable proportion of high genetic merit breeding bulls produce poor-quality semen, the transcriptomic alterations in spermatozoa from such bulls are not understood. In the present study, comparative high-throughput transcriptomic profiling of spermatozoa from good and poor-quality semen-producing bulls was carried out to identify the transcripts associated with semen quality. Using next-generation sequencing (NGS), we identified 11,632 transcripts in Holstein Friesian bull spermatozoa; after total hit normalization, a total of 544 transcripts were detected, of which 185 transcripts were common to both good and poor-quality semen, while 181 sperm transcripts were unique to good quality semen, and 178 transcripts were unique to poor-quality semen. Among the co-expressed transcripts, 31 were upregulated, while 108 were downregulated, and 46 were neutrally expressed in poor-quality semen. Bioinformatics analysis revealed that the dysregulated transcripts were predominantly involved in molecular function, such as olfactory receptor activity and odor binding, and in biological process, such as detection of chemical stimulus involved in sensory perception, sensory perception of smell, signal transduction, and signal synaptic transmission. Since a majority of the dysregulated transcripts were involved in the olfactory pathway (85% of enriched dysregulated genes were involved in this pathway), the expression of selected five transcripts associated with this pathway (OR2T11, OR10S1, ORIL3, OR5M11, and PRRX1) were validated using real-time qPCR, and it was found that their transcriptional abundance followed the same trend as observed in NGS; the sperm transcriptional abundance of OR2T11 and OR10S1 differed significantly (*p* < 0.05) between good and poor-quality semen. It is concluded that poor-quality semen showed altered expression of transcripts associated with olfactory receptors and pathways indicating the relationship between olfactory pathway and semen quality in bulls.

## Introduction

Globally, for genetic improvement and enhancing productivity, artificial insemination (AI) of cows with high-genetic merit bulls is being carried out. Holstein Friesian bulls are used for crossbreeding programs with local breeds/non-descript cattle in many parts of the tropical/subtropical countries. Although AI of native cows with cryopreserved spermatozoa from high genetic exotic bulls/crossbred bulls improved the milk production substantially, the coverage of AI is limited in these countries. One of the major reasons for the low coverage of AI is the limited availability of quality semen (1). Generally, breeding bulls are selected based on their genetic merit and their ability to fulfill the requirements of breeding soundness evaluation (BSE). The bulls are subjected to assessment of the genital tract, accessory organs, scrotal circumference, and semen quality parameters, such as motility, sperm morphology, and concentration of spermatozoa as a part of BSE (2). However, a significant proportion of bulls qualified through the BSE are culled because of poor-quality semen and subfertility during later stages. Furthermore, 20–25% variation in conception rates was observed among the bulls that qualified through the BSE (3, 4), and a substantial proportion of bulls that have passed the BSE had high ejaculation rejection rates (5, 6). Ejaculate rejection rate indicates the number of ejaculates rejected from being used in AI (because of poor semen quality) from a bull over a period of time. It has been reported that ejaculate rejection rate in breeding bulls ranged from 10 to 100% with the average being 52 to 55% (7–9). High ejaculate rejection rates lead to colossal loss to the breeders and hamper genetic improvement.

Although the incidence of inferior semen quality in breeding bulls has been documented in detail, the etiology remains elusive. The ejaculates are rejected for various reasons including poor mass activity, less concentration, initial motility, and poor progressive motility. Several reasons have been attributed to poor semen quality in breeding bulls including pathologies of testicular origin (10, 11), maturational deficiencies (12), alterations in seminal plasma constituents (13), and molecular alterations (14–16). These studies collectively indicate alterations in sperm phenotypic or functional attributes in poor-quality semen; however, it has been shown that assessment of a few sperm phenotypic characteristics may increase the accuracy of selection of bulls but cannot precisely predict the bull performance. Therefore, assessment of the molecules present in the spermatozoa assumes significance because it provides information about the spermatozoa, testicular health, the process of spermatogenesis, and sperm maturation. Sperm RNA has been shown to have a role in spermatogenesis, sperm maturation, and sperm function (14). Earlier, it was believed that sperm just act as a transporter of paternal genome. Of late, it is well documented that spermatozoa, besides carrying the paternal genome, harbors several molecules including different types of RNAs. Although research on sperm RNA started many years ago, the first reports on sperm transcriptome profiling in human (17), bovine (18), and equine (19) were published recently. Very recently, a study reported the transcriptomic profile of bull spermatozoa that suggested possible implications of transcriptomic variations on semen quality and fertility (20).

Mature spermatozoa are highly differentiated cells devoid of transcription and translation activity; however, ejaculated spermatozoa retain a pool of complex and specific RNAs. These spermatozoal RNAs have a role in spermatogenesis, chromatin repackaging, oocyte activation, genomic imprinting, early embryonic development, epigenetic modification, and placental development (14, 19). A considerable number of studies have been performed to decrypt the profile of sperm mRNA population by utilizing various methods, for example, microarrays (15, 21–24), suppression subtractive hybridization (25, 26), and next-generation RNA sequencing (16, 20, 27–29). Next-generation sequencing technology to study the transcriptomic profile of spermatozoa is a reliable technique to understand molecular aspects of semen quality, which gives information not only about the ejaculated semen but also about the past events starting from spermatogenesis. Since poor semen quality is one of the major factors affecting the lifetime frozen semen dose production from a breeding bull, there is a need to understand the underlying etiology. With the developments in molecular tools, it is possible now to study the molecules present in the spermatozoa and how it is altered in poor-quality semen-producing breeding bulls. Once the molecular alterations are identified, it would be possible for us to evolve measures for sperm quality improvement. With this backdrop, the present study was undertaken (i) to profile the transcripts in spermatozoa derived from good and poor-quality semen-producing bulls using a high-throughput NGS platform, and (ii) to compare the sperm transcriptomic profile between good and poor semen-producing bulls for identification of the sperm transcripts associated with semen quality.

## Materials and Methods

### Experimental Bulls

The present investigation was carried out at the Theriogenology Laboratory, Southern Regional Station of ICAR-National Dairy Research Institute, Karnataka, India. All the experimental procedures were duly approved by the Institute Animal Ethics Committee (Approval No: 1904/GO/ReBi/L/16/CPCSEA) and performed in accordance with relevant guidelines and regulations. Semen production details of Holstein Friesian breeding bulls (*n* = 50) were assessed over a period of 1 year. All the breeding bulls successfully underwent breeding soundness evaluation and were routinely used in AI. These bulls were maintained under uniform management conditions including housing, feeding, and health care measures as per the minimum standard protocol for breeding bull management (MSP, Government of India). For each bull, the number of ejaculates rejected for subsequent processing and cryopreservation, owing to poor initial semen quality, was recorded. Only those ejaculates fulfilling standard requirements (>600 million spermatozoa/ml; >0% progressive motility and <20% sperm abnormalities) were considered as fit for cryopreservation and AI. Other ejaculates were rejected from subsequent cryopreservation and use in AI. The ejaculate rejection rate was calculated based on the following formula:

Ejaculate rejection rate (ERR) (%) = [(total number of ejaculate rejected/total number of ejaculate collected) × 100]

The ejaculate rejection rates of all the 50 bulls used for the study are shown in Supplementary Table S1. Based on the ejaculate rejection rate, a total of 12 bulls were selected for the study. Six bulls had very high ejaculate rejection rate (>60% rejection, i.e., >60% of ejaculates produced were of poor quality), and the remaining six bulls had very low ERR (<5%, i.e., only >95% of ejaculates were of good quality), and the difference was highly significant (p < 0.01). Ejaculates were collected from the bulls using artificial vagina as per the standard procedure, and fresh spermatozoa were used for transcriptomic profiling.

### RNA Extraction From Spermatozoa

For RNA extraction, pure sperm fraction obtained using 90–45% discontinuous Percoll gradient was used. This procedure eliminated contaminating substances like epithelial cells and seminal plasma. Total RNA was extracted from sperm using TRIzol (Ambion, Thermo Fisher Scientific, USA) as previously described (30) with minor modifications. The fresh semen samples from experimental bulls were centrifuged at 800 × *g* for 10 min at 4°C, and the supernatant was removed without disturbing the sperm pellet, and the pellet was washed twice with 1 ml of DPBS at 800 × *g* for 15 min at 4°C. The sperm pellet was further purified by mixing with 1 ml of somatic cell lysis buffer (0.1% SDS, 0.5% Triton X-100 in DEPC treated water) and kept over ice for 1 h with consistent vertexing for every 15 min and centrifuged (5,939 × *g* for 1 h at 4°C). The pellet was then dissolved in 1 ml of DEPC-treated water and centrifuged again (5,939 × *g* for 15 min at 4°C). The washed pellet was suspended in 1 ml of prewarmed TRIzol reagent (65°C) and shredded by sonication process for 1 min and 30 s (shredding for 6 s and break for 5 s). The samples were then vortexed for 5 min and incubated in dry bath (62°C) for 1 h for complete dissociation of the sperm membrane. To the lysate, 200 μl of chloroform was added, mixed vigorously for 20 s, and allowed to stand without disturbance at room temperature for 15 min. The mixture was centrifuged (13,362 × *g*, 4°C, 20 min) without applying a break to separate the phases. Following centrifugation, three layers were formed: the clear aqueous layer containing RNA, the opaque white interphase containing DNA, and the red bottom organic layer containing protein. The upper aqueous layer containing RNA was carefully aspirated without disturbing the interphase and transferred to another tube. Then isopropanol (0.5 ml) was added and gently mixed by inverting the tubes. The mixture was then incubated at room temperature for 15 min and centrifuged (13,362 × *g* for 15 min at 4°C). The supernatant was discarded, and 1 ml of 70% ethanol was added to the RNA pellet and centrifuged at 13,362 × *g* for 10 min. Ethanol was removed, and the RNA pellet was air dried to remove traces of ethanol. The pellet was dissolved in 20–40 μl of DEPC treated water and the isolated RNA was subjected to DNase treatment using a TURBO DNA- free Kit (Thermo Fisher Scientific, USA) according to the instructions of the manufacturer. The RNA quantification was performed by spectrophotometry using Nanodrop (ND-1000, Thermo-Scientific, USA). RNA samples in equal quantity were pooled from good and poor-quality semen for further analysis.

### Transcriptome Library Preparation

Total RNA (1 μg) was used to enrich mRNA using NEB Magnetic mRNA Isolation Kit (Illumina, USA). The transcriptome library was prepared by using NEB ultraII RNA Library Prep Kit (Illumina, USA) and sequenced using Illumina Next Seq 500 (Illumina, USA) paired-end technology. The enriched mRNA was fragmented (approximately 200 bp) using fragmentation buffer. Random hexamer primers were added and hybridized to complementary RNA sequences. These short fragments were used as templates to synthesize the first-strand cDNA using reverse transcriptase and dNTPs. The DNA–RNA hybrids synthesized during first-strand cDNA synthesis were converted into full-length double-stranded cDNAs using RNase H and *E. coli* DNA polymerase I, and the second strand cDNA was synthesized using second-strand enzyme mix and buffer. The double-stranded cDNA fragments were purified using 1.8 × Ampure beads. The purified double-stranded cDNA was end-repaired to ensure that each molecule is free of overhangs and has 5′ phosphates and 3′ hydroxyls before the adaptor ligation. The adaptor-ligated DNA was purified using Ampure beads and enriched with specific primers, compatible for sequencing on the Illumina platforms. The final enriched library was purified and quantified by Qubit ® 3.0 Fluorometer (Thermo Fisher Scientific, USA), and the size was analyzed by 2100 Bioanalyzer (Agilent, USA).

### Sperm RNA Sequencing and Data Analysis

The cDNAs synthesized samples of good quality semen and poor-quality semen were sequenced using Illumina Next seq-500 sequencing system (Genotypic Technology, Bengaluru, India) to generate paired-end 76-bp reads. The sequence analysis was performed using the online server tool Galaxy (https://usegalaxy.org/) (31). Raw data generated from the two sperm samples and read quality were checked using FastQC (Galaxy version 0.72) program, and the reads of raw data were processed with Cutadapt tool (Galaxy Version 1.18) (32). Processing includes removal of adapter (AGATCGGAAGA) sequence, length filter (>15 bp), and quality trimming (30 Phred score). Using HISAT2 (Galaxy Version 2.1.0+galaxy4) (33) tool the two-sample high-quality processed reads were aligned to the bovine genome (*Bos taurus* UCD 1.2), and the samples were sorted with aligned sequences using Samtools (Galaxy Version 2.0.2) (34). The mapped and properly paired sequence to the reference genome was calculated based on tabular descriptive statistics dataset tool Flagstat (Galaxy Version 2.0.1) (34). Using the tool Cufflinks (Galaxy Version 2.2.1.2) (35), the presence of individual transcripts and their expression levels were normalized. Normalization was carried out for differential expression study, since there are differences in total number of reads in each sample. The normalized count is required for comparison across the samples; therefore, the normalized count score for each sample is calculated as fragments per kilobase of exon per million fragments mapped (FPKM). [FPKM = (Rmg × 10^6^)/(Rmt × L); Rmg = the number of reads mapped to per gene/transcript; Rmt = the total number of reads mapped to protein coding sequence in the alignment (overall alignment); L = the length of the exon in bp]. The data of cufflink assembly were merged using Cuffmerge (Galaxy Version 2.2.1.2). The Cuffdiff (galaxy version 2.2.1.5) was used to identify significant changes in transcripts expression, splicing, and promoter between good and poor semen quality groups. Cuffdiff returns a *p*-value of 5E−05 as the minimum. It involves 20,000 replicates, and the counts of genes having extreme values with a single pseudo-count (1/20,000 = 5E-05). Anything below the extreme value receives a *p*-value of 5E−05.

The transcripts were categorized as differentially expressed transcripts based on log2 fold changes as follows: fold change > +1 (upregulated in poor-quality semen), <-1 (downregulated in poor-quality semen) and between < +1 and >-1 (neutrally regulated between good and poor-quality semen). The transcripts with FPKM present only in the good quality semen were considered as unique to bulls producing good quality semen, while transcripts with FPKM present only in the poor-quality semen were considered as unique to bulls producing poor-quality semen.

The total number of transcripts were plotted using the online tool Venny (Version 2.1.0). The neutrally and differentially expressed sperm transcripts between good and poor-quality semen were plotted using Volcano plot based on the transcript log2 (fold change) and *p*-value. A heatmap was generated for the selected differentially expressed sperm transcripts between good and poor-quality semen groups using Clustvis (36).

### Annotation of Genes Detected by RNA Sequencing

Gene ontology (GO) and functional annotation of sperm transcripts were analyzed using Uniport (https://www.uniprot.org/) and the Database for Annotation, Visualization, and Integrated Discovery (DAVID) Bioinformatics Resources (v6.8) (https://david.ncifcrf.gov/) into three main categories: the molecular function (MP), biological process (BP), cellular component (CC) and Kyoto Encyclopedia of Genes and Genomes (KEGG) pathway analysis. The top 10 biological processes, cellular components, and molecular functions were plotted as Donut pie chart using Highcharts (https://www.highcharts.com/demo/pie-donut). Pathway enrichment was plotted out using R Library Custer Profiler, and pathway enrichment graph was carried using enrich KEGG functions. Interaction of genes and detailed network analysis of combined gene ontology categories and pathway analysis was performed using ClueGo (Version 2.5.4) plugins in the open-source Cytoscape (Version 3.7.1) (Cluego.org) platform (37). All the analyses were performed with *Bos taurus* as background.

### Validation of Selected Transcripts by qPCR

The *in silico* analysis helped in identifying the differentially expressed genes between good and poor-quality semen, among which five genes (*OR1L3, OR5M11, PRRX1, OR2T11*, and *OR10S1*) were selected based on their log2 fold change value and their involvement in pathways related to olfactory transduction for the validation in all the 12 bulls (good = 6; poor = 6) using qPCR. The primer sequence, product size, and annealing temperature are given in Table 1. Primers were designed using the web-based software PRIMER-3 across exon–exon junctions in order to eliminate contaminating genomic DNA amplification. The extracted RNA (as mentioned earlier) was converted into cDNA by using RevertAid First Strand cDNA Synthesis Kit (Thermo Fisher Scientific, USA). The annealing temperatures of primers for the selected genes were optimized using PCR (Prima-96 plus, Himedia), and the cDNAs prepared from different samples were subjected to qPCR analysis. Real-time quantitative PCR was performed on Insta Q96 Plus Real Time Machine PCR system (HiMedia, India) in a 15-μl reaction comprising 1 μl of cDNA, 0.25 μl (10 pmol/μl) of forward and reverse primers, and 7.5 μl of Maxima SYBR Green/ROX qPCR master mix 2×. The thermal cycling conditions consisted of initial denaturation at 95°C for 10 min, followed by 40 cycles of 95°C for 15 s, 60°C for 30 s, and 72°C for 30 s. Relative gene expression levels were determined using the 2^−ΔΔCt^ method, where ΔCt = Ct target – Ct internal reference and ΔΔCt = ΔCt _target_ – ΔCt _calibrator_. *GAPDH* served as the internal reference gene. Gene expression data were normalized against *GAPDH* expression. The calibrator in each study consisted of cDNA from the corresponding control group. Relative mRNA expression is expressed as n-fold mRNA expression relative to the calibrator. The differences in fold change values between two groups were evaluated by *t*-test using SPSS (22.0, IBM, USA) software. The difference was considered as significant when p < 0.05. The specificity and integrity of the PCR products were ensured by melting curve analysis, whereas the appropriateness of size was confirmed by agarose gel electrophoresis. The experiment was repeated thrice, each time in duplicate.

**Table 1:** Details of primers used for real-time expression analysis.

**S. no**	**Genes**	**Primer sequences**	**Product size**	**Annealing temperature**
1	*GAPDH*	FP-CTGAGGACCAGGTTGTCTCCTG	141 bp	60°C
		RP-CCCTGTTGCTGTAGCCAAATTC		
2	*ORIL3*	FP-GGCCTTCTCCACAGCTTTCT	181 bp	60°C
		RP-AACCAGCCCTTCTGTCATGG		
3	*OR5M11*	FP-ATACCTCTACTGCAGCCCCA	153 bp	60 °C
		RP-CGTAGCGGTCATAGGCCATT		
4	*PRRX1*	FP-CATCGTACCTCGTCCTGCTC	150 bp	60°C
		RP-GTAGCCATGGCGCTTTTCAG		
5	*OR2T11*	FP-GCTCCTGGTGGACATGGTTT	187 bp	60°C
		RP-GAGACACACTCTGCGGTTCA		
6	*OR10S1*	FP-GCTGGGATCACTTGGACCAT	98 bp	60°C
		RP-AAGTAGGCGATGTGACGAGG		

## Results

Bio-analyzer analysis of spermatozoal RNA isolated from the bull spermatozoa showed the lack of 18s and 28s rRNA peaks in sperm (Supplementary Figures S1A,B), indicating that the total RNA isolated from spermatozoa had no contamination from somatic cells, leukocytes, testicular germ cells, and other cells.

Using Illumina Next Seq-500 RNA sequencing platforms, we identified 15,028,305 and 15,373,636 with paired-end 76 bp raw reads in spermatozoa of good and poor-quality semen, respectively. After processing of raw reads, processed reads in good quality semen and poor-quality semen were 14,499,211 and 14,981,485 reads, respectively. The processed reads were mapped to the reference genome (*Bos taurus*), which showed an alignment of ≃73% and ≃83% for good and poor-quality semen, respectively. A total of 11,632 transcripts were identified in Holstein Friesian bull spermatozoa, in which 5,673 transcripts were detected in good and 9,004 transcripts were detected in poor-quality semen, while 3,045 transcripts were common to both. After total hit normalization, a total of 544 transcripts were detected, of which 185 transcripts were common to both good and poor-quality semen, while 181 and 178 transcripts were unique to good and poor-quality semen, respectively. Among the co-expressed genes (185), 31 genes were upregulated (>1-fold), 108 genes were downregulated (>-1-fold), whereas 46 genes were neutrally expressed (<−1 to 1) in poor-quality semen (Supplementary Figure S2). The top 10 upregulated and downregulated transcripts in poor compared with good quality semen are listed in Tables 2, 3, respectively. Top 10 abundantly expressed sperm transcripts unique to good and poor-quality semen are given in Tables 4, 5, respectively.

**Table 2:** The top 10 upregulated transcripts in poor-quality semen compared with good quality semen.

**S. no**	**Transcripts**	**Description**	**FPKM**	**FPKM**	**log2**	***p*-Value**
			**(good quality)**	**(poor quality)**	**(fold_change)**	
1	HBEGF	Heparin binding EGF like growth factor	0.06	0.91	3.71	0.27
2	CPN2	Carboxypeptidase N subunit 2	0.10	1.06	3.32	0.10
3	SYNPO2L	Synaptopodin 2 like	0.20	1.08	2.42	0.19
4	MUC15	Mucin 15, cell surface associated	0.27	0.98	1.86	0.44
5	CSMD3	CUB and Sushi multiple domains 3	0.29	1.02	1.80	0.16
6	PRRX1	Paired related homeobox 1	0.55	1.70	1.61	0.38
7	OR2T11	Olfactory receptor family 2 subfamily T member 11	0.41	1.08	1.39	0.32
8	ND4	NADH dehydrogenase subunit 4	0.41	1.09	1.39	0.47
9	ONECUT1	One cut homeobox 1	0.37	0.91	1.30	0.42
10	OR10S1	Olfactory receptor family 10 subfamily S member 1	0.65	1.43	1.13	0.61

*FPKM, fragments per kilobase of exon per million fragments mapped*.

**Table 3:** Top 10 downregulated transcripts in poor compared with good quality semen.

**S. no**	**Transcripts**	**Description**	**FPKM**	**FPKM**	**log2**	***p*-Value**
			**(good quality)**	**(poor quality)**	**(fold_change)**	
1	SALL2	Spalt like transcription factor 2	1.28	6.59613E−05	−14.24	0.20
2	CLEC7A	C-type lectin domain containing 7A	1.18	6.39692E−05	−14.18	0.13
3	CDH19	Cadherin 19	0.94	0.05	−4.00	0.25
4	USP31	Ubiquitin specific peptidase 31	1.08	0.07	−3.81	0.22
5	RGS2	Regulator of G protein signaling 2	2.60	0.20	−3.64	0.28
6	OR1L3	Olfactory receptor family 1 subfamily L member 3	1.15	0.11	−3.36	0.27
7	POU2AF1	POU class 2 homeobox associating factor 1	1.00	0.10	−3.31	0.05
8	VSTM5	V-set and transmembrane domain containing 5	1.79	0.19	−3.18	0.30
9	LMO4	LIM domain only 4	1.09	0.12	−3.12	0.21
10	DLK1	Delta like non-canonical Notch ligand 1	1.37	0.17	−3.00	0.06

**Table 4:** Top 10 abundantly expressed transcripts unique to good quality semen.

**S. no**	**Transcripts**	**Description**	**FPKM**	***p*-Value**
1	U8	Small nucleolar RNA	39.21	0.04
2	RPS29	Ribosomal protein S29	5.34	0.04
3	RNASE1	Ribonuclease A family member 1, pancreatic	4.74	0.12
4	KRTAP15−1	Keratin associated protein 15−1	4.52	0.01
5	Telomerase -vert	Telomerase-vert	1.84	0.04
6	TDRP	Testis development related protein	1.41	0.04
7	RPS27L	Ribosomal protein S27 like	1.31	0.02
8	ZMAT3	Zinc finger matrin-type 3	1.15	0.01
9	OR4N2	Olfactory receptor family 4 subfamily N member 2	1.10	0.04
10	FAM131A	Family with sequence similarity 131 member A	0.90	0.04

**Table 5:** Top 10 abundantly expressed transcripts unique to poor-quality semen.

**S.no**	**Transcripts**	**Description**	**FPKM**	***p*-Value**
1	7SK	RNA component of 7SK nuclear ribonucleoprotein	3.62	0.04
2	ARF6	ADP ribosylation factor 6	2.27	0.03
3	SIX1	SIX homeobox 1	1.70	0.00
4	MSGN1	Mesogenin 1	1.44	0.04
5	OR4E2	Olfactory receptor family 4 subfamily E member 2	1.41	0.00
6	OR1B1	Olfactory receptor family 1 subfamily B member 1	1.04	0.04
7	OR11H6	Olfactory receptor family 11 subfamily H member 6	1.00	0.04
8	OTP	Orthopedia homeobox	0.98	0.00
9	KRTAP27-1	Keratin associated protein 27-1	0.90	0.00
10	SNRNP27	Small nuclear ribonucleoprotein U4/U6. U5 subunit 27	0.90	0.04

**Figure 1:**
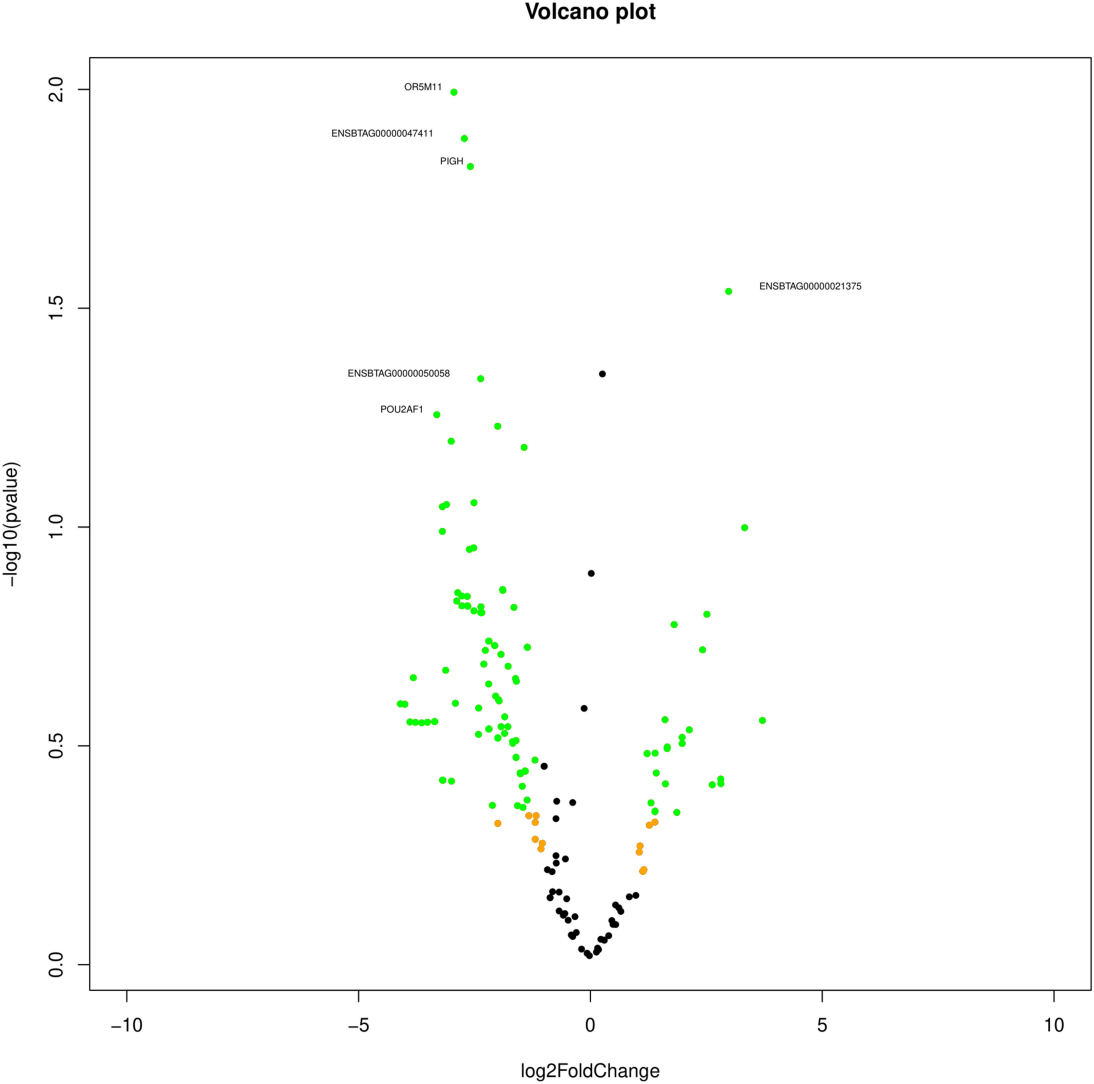
Volcano plot indicating the differentially expressed transcripts between good and poor semen-quality bulls.

Based on the transcript log 2 (fold change) and *p*-value (<0.05), significance, the neutrals and differentially expressed transcripts are plotted in the Volcano plot (Figure 1). The transcript ENSBTAG00000021375 was upregulated, while olfactory receptor family 5 subfamily M member 11 (*OR5M11*), ENSBTAG00000047411, phosphatidylinositol glycan anchor biosynthesis class H (*PIGH*), ENSBTAG00000047411, POU class 2 homeobox-associating factor 1 (*POU2AF1*), C type domain-containing 7A (*CLEC7A*), and Spalt-like transcription factor 2 (*SALL2*) were downregulated in poor-quality semen.

### Functional Annotation of Dysregulated Genes in Inferior Poor Quality

#### Biological Process

**Figure 2:**
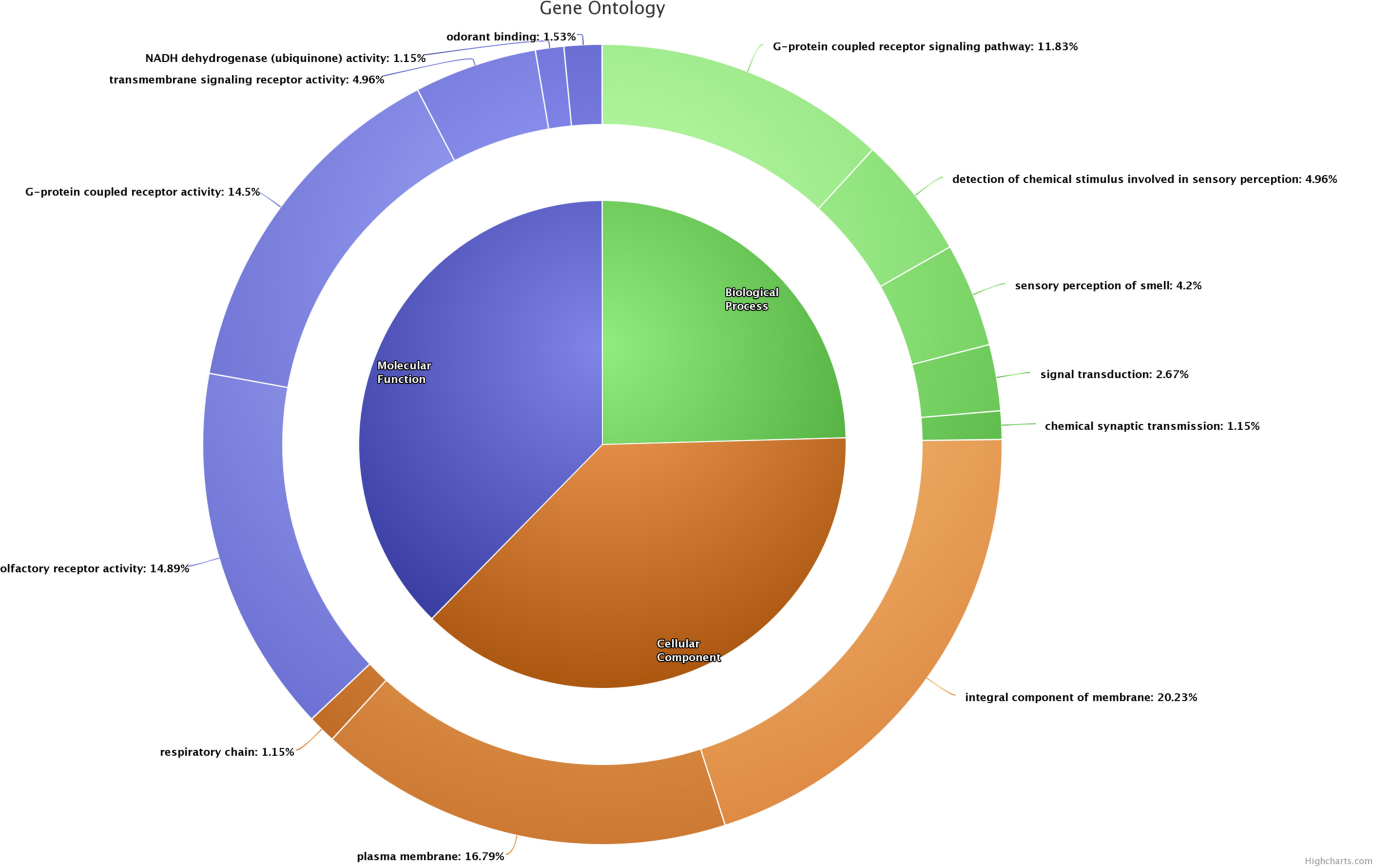
Functional classification (biological process, cellular component, and molecular function) of dysregulated transcripts based on gene ontology.

In poor-quality semen, a majority of the dysregulated genes (*n* = 31) were involved in G-protein-coupled receptor signaling pathway (GO:0007186), in which *ORT2T11* (olfactory receptor, family 2, subfamily T, member 11) was the known upregulated gene, whereas the remaining known genes were downregulated. In addition, dysregulated genes were involved in olfactory-related biological process such as detection of chemical stimulus involved in sensory perception (GO:0050907), sensory perception of smell (GO:0007608), signal transduction (GO:0007165), and chemical synaptic transmission (GO:0007268). Functional annotation of (biological process, cellular component, molecular function) dysregulated transcripts based on gene ontology (GO) are presented in Figure 2.

#### Cellular Component

A total of 53 dysregulated genes in poor-quality semen were related to the integral component of membrane (GO:0016021). Among these, five genes [*OR2T11* (olfactory receptor, family 2, subfamily T, member 11), *MUC15* (mucin 15, cell surface associated), *ND4* (NADH dehydrogenase subunit 4), *CYTB* (cytochrome b), *CSMD3* (CUB and Sushi multiple domains 3)] were upregulated in poor-quality semen. A total of 44 dysregulated genes were related to the plasma membrane (GO:0005886), in which two known genes (*OR2T11* and *MUC15*) were upregulated, while the remaining known genes were downregulated in poor-quality semen. The three dysregulated genes (*ND3, CYTB* and *ND6*) were related to the respiratory chain (GO:0070469), in which *CYTB* was upregulated, while *ND3* and *ND6* were downregulated in poor-quality semen.

#### Molecular Function

Majority of the dysregulated genes were involved in olfactory receptor activity (GO:0004984) and G-protein-coupled receptor activity (GO:0004930), among which *OR2T11* was the only known upregulated gene involved in both of these functions, and the remaining genes involved in these molecular functions were downregulated. A considerable number of genes were involved in molecular functions such as G-protein-coupled receptor activity (GO:0004930), and NADH dehydrogenase (ubiquinone) activity (GO:0008137), and odorant binding (GO:0005549), respectively.

### Pathway Enrichment of Dysregulated Transcripts in Poor-Quality Semen

**Figure 3:**
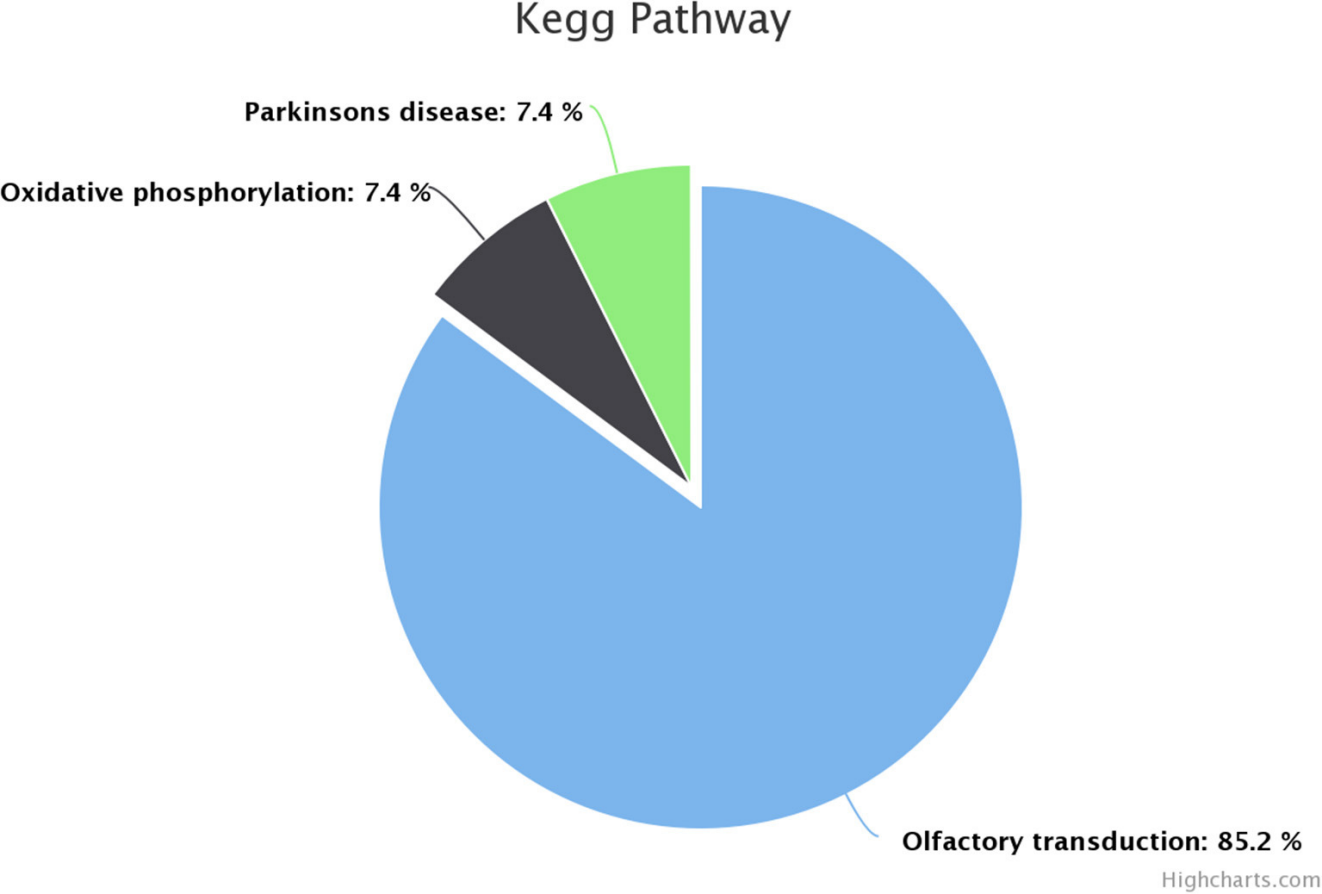
Kyoto Encyclopedia of Genes and Genomes (KEGG) pathway enrichment of dysregulated genes.

**Figure 4:**
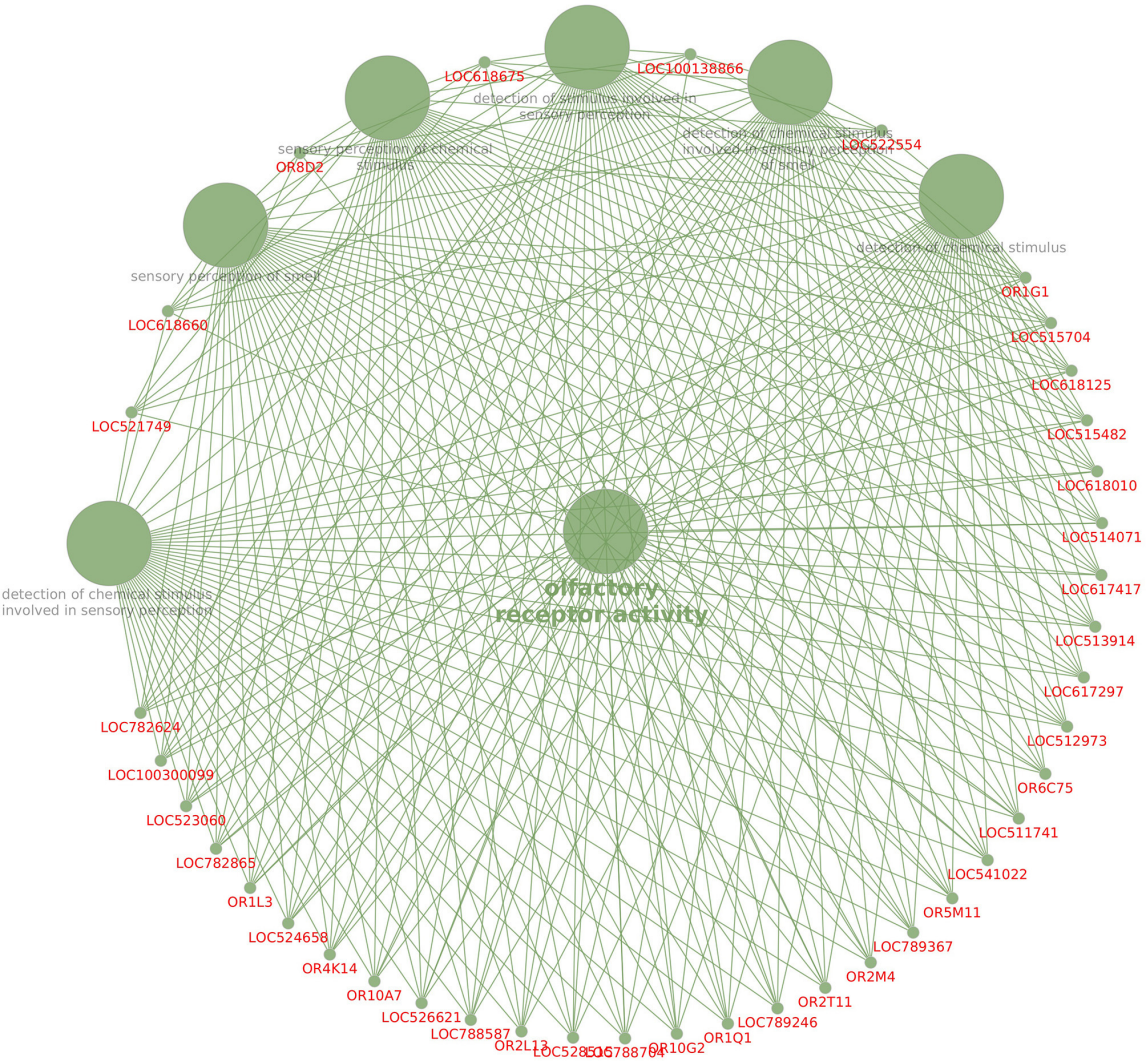
Interaction between the dysregulated genes involved in the olfactory pathway.

KEGG pathway analysis revealed that dysregulated genes in poor-quality semen were involved in three pathways (Figure 3), among which 85.2% of the genes (*n* = 46) were involved in olfactory transduction pathway (bta04740; *p*-value 6.03E−18). The interaction between the genes involved in olfactory transduction pathway in our study is shown in Figure 4. Some of the dysregulated genes were involved in the oxidative phosphorylation (bta00190; *p*-value 0.058) and Parkinson's disease (bta05012; *p*-value 0.071) pathways. The genes involved in both of these pathways are of the same group (*CYTB, ND6, ND4*, and *ND3*). Among these genes, *CYTB* and *ND4* were upregulated, while *ND6* and *ND3* were downregulated in poor-quality semen.

### Real-Time Expression Analysis of Select Genes

**Figure 5:**
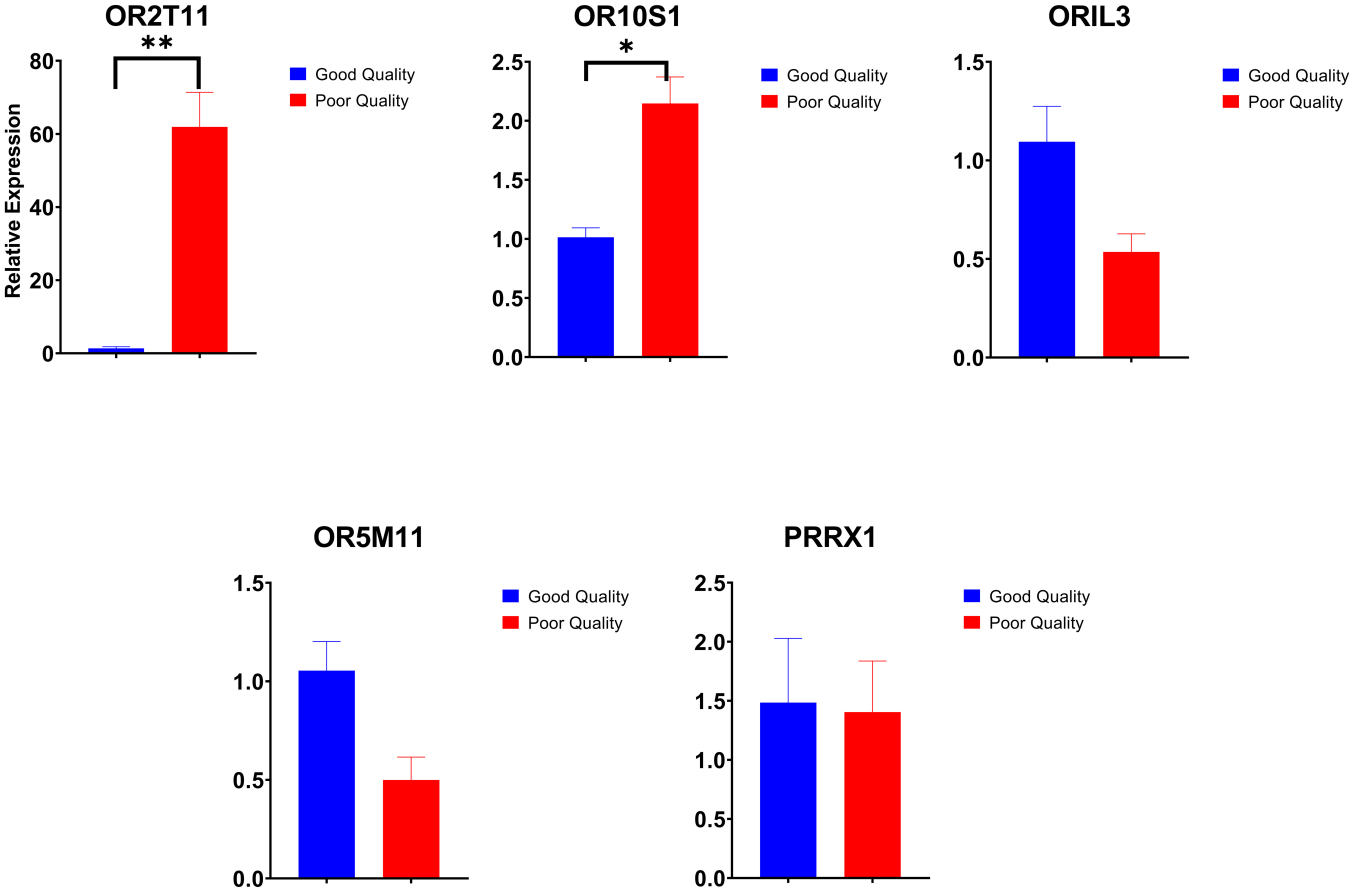
Relative expression of selected genes in good and poor-quality semen analyzed by real-time qPCR (*p < 0.05 and **p < 0.01).

Among the dysregulated genes, five genes (*OR2T11, OR10S1, OR1L3, OR5M11*, and *PRRX1*) were selected based on fold change for qPCR expression analysis. Among the five genes selected for validation, three genes were upregulated, and two genes were downregulated in NGS data of poor-quality semen. All these five genes were validated by qPCR analysis, and the validation results followed the same trend as observed in NGS analysis. The results of qPCR expression analysis of selected genes are presented in Figure 5. Among the five genes, two genes (*OR2T11, OR10S1*) were significantly upregulated (*p* < 0.05) in poor-quality semen as per qPCR results. Dysregulation (log2 fold change) of *OR10S1* was 1.13 in NGS and 1.07 in qPCR, but the *OR2T11* was 1.39-fold in NGS but 5.90 in qPCR. The transcriptional abundance of *ORIL3, OR5M11*, and *PRRX1* were downregulated in poor-quality semen; however, the difference was not enough to be statistically significant.

## Discussion

Using high-throughput NGS platform, we identified a total of 11,632 transcripts in Holstein Friesian bull spermatozoa, which is comparable with earlier findings that reported the number of transcripts detected in bull spermatozoa varied from 3,227 to 14,711 (20, 29, 38, 39). The total number of transcripts observed in bull spermatozoa varied among studies, which might be due to differences in season of semen collection, fresh or cryopreserved semen, RNA isolation methods (30), integrity of sperm RNA, RNA-sequencing instrument (40), and library preparation methods (41).

### Unique Transcripts to Good and Poor-Quality Semen

Among the 10 abundantly expressed transcripts unique to good quality semen, small nucleolar RNA (*U8*) was very highly expressed. It is a conserved, abundant, short noncoding RNA, which regulates the methylation and pseudo-uridylation of various classes of RNA (42). The remaining transcripts have been shown to be involved in spermatogenesis, sperm maturation, and functions. Ribonuclease A family member 1, pancreatic (*RNASE1*) secreted by epididymis is involved in host defense mechanisms of the male reproductive tract, sperm maturation, and male fertility (42, 43). Testis development-related genes (*TDRP*) regulates the testicular functions, steroidogenesis, and spermatogenesis process (44). Disturbances in ribosomal protein S27 (*RPS27L*) causes apoptosis, depletion of bone marrow, and genomic instability (45). Zinc finger matrin-type 3 (*ZMAT3*) gene contributes to the TP53-mediated apoptosis (46). Among the 10 abundantly expressed transcripts unique to poor-quality semen, the RNA component of 7SK nuclear ribonucleoprotein (*7SK*) regulate the activity of kinase-positive transcription elongation factor (47). *ARF6* gene enhances the sperm phospholipase D activity to produce phosphatidic acid and stimulates the synthesis of phosphatidylinositol 4,5-bisphosphate, and also has a role in fertilization (48). SIX homeobox 1 (*SIX1*) gene was reported to be involved in embryogenesis, cellular specification, and postnatal gonad development in mammals. *OR2BI is* a G-protein-coupled receptor and involved in liver cell metabolism (49). Orthopedia homeobox *(OTP*) was used as a potential marker for lung carcinoids in humans (50).

### Top 10 Differentially Expressed Transcripts Between Good and Poor-Quality Semen

Among the top 10 upregulated transcripts in poor-quality semen, heparin-binding EGF-like growth factor (*HBEGF*) was the most upregulated transcript with 3.71-fold change in poor compared with good quality semen. It is reported that *HBEGF* induces sperm membrane changes in the female oviduct environment and thereby involved in sperm capacitation, acrosome reaction (51), formation of the oviduct reservoir (52), and sperm-oocyte binding (53). Carboxypeptidase N subunit 2 was involved in the innate immunity of the male reproductive tract and also controls the sperm fertilization process by interacting with heparin molecules (54). Synaptopodin 2-like transcript (*SYNPO2L*) is an indicator of terminal differentiation of cells and is involved in maintaining cell morphology (55). CUB and Sushi multiple domains 3 (*CSMD3*) was highly expressed in human testis and had a potential role in male fertility, brain development, and function (56). Olfactory receptor family 2 subfamily T member 11 (*OR2T11*) is the odorant molecule that is responsive to thiol (57, 58). The association of NADH dehydrogenase subunit 4 (*ND4*) transcripts with sperm motility, semen quality, and male infertility in Chinese human population is involved in enhancing the ATP synthase activity and energy demand required for sperm motility (59). One cut homeobox 1 (also known as HNF-6) plays a role in cellular processes that includes glucose metabolism and regulation of cell cycle (60).

Among the top 10 downregulated sperm transcripts in poor-quality semen, Spalt-like transcription factor 2 (*SALL2*) was the most downregulated (-14.24-fold change) in comparison with good quality semen. It has a role in neurogenesis, neuronal differentiation, and eye development (61–63). C-type lectin domain-containing 7A gene is involved in inflammation, immune response, and recognition of viral and bacterial pathogens (64). Adherins are calcium-dependent molecules involved in embryonic development and the regulation of morphogenesis (65). Ubiquitin-specific peptidase 31 transcript was abundantly expressed in spermatogonia, and its expression decreases during meiotic and pachytene cell division (66–68). Regulator of G protein signaling 2 (*RGS2*) gene suppresses the premature calcium release in mouse eggs (69). POU class 2 homeobox associating factor 1 regulates B-cell development and is expressed in the leukocytes of mice (70). V-set and transmembrane domain-containing 5 (*VSTM5*) gene involved in the regulation of phagocytes in humans (71). LIM domain only 4 (*LMO4*) plays a role in cell fate (72, 73) and negative regulation of estrogen receptor transactivation (74). Delta-like non-canonical Notch ligand gene was involved in the regulation of mosquito reproduction and fertility (75) and in the development of bovine embryos (76).

### Possible Reasons for Poor Semen Quality

#### Altered Sperm Maturation Processes and Plasma Membrane Integrity

G-protein-coupled receptors (GPCR) are the activators of conventional adenylyl cyclase (CAC) to generate cAMP (77) and have a role in sperm capacitation, actin polymerization, and acrosome reaction (78, 79).Under normal conditions, the female reproductive tract contains GPCR activators, such as ouabain, angiotensin II, EGF, and lysophosphatidic acid, which can activate the epidermal growth factor receptor (EGFR) during the final stages of capacitation to increase the intracellular calcium concentration, thereby resulting in F-actin breakdown and the process of acrosome reaction (80, 81). We observed that expression of 32 genes involved in G-protein-coupled receptor signaling pathway (GO:0007186) was dysregulated in poor-quality semen, which might be related to the alterations in sperm maturation processes such as capacitation and acrosome reaction. The functional annotation of dysregulated genes based on cellular components indicated the 44 dysregulated genes, which are the vital machinery of the plasma membrane (GO:0005886). The sperm membrane is the important component, whose integrity is essential for sperm viability, and its functionality is vital for the sperm-oviduct epithelium binding or the sperm-oocyte interaction during fertilization (82, 83). Therefore, the dysregulation of genes related to the plasma membrane might be related to the impaired plasma membrane integrity in the sperm of bulls producing poor-quality semen. Earlier findings on altered sperm membrane integrity in low-fertile bulls (84, 85) support our hypothesis.

#### Unhinged Mitochondrial Energy Production

The genes involved in the respiratory chain are important for sperm mitochondrial activity and sperm motility (86). We observed that three genes (*ND3, CYTB*, and *ND6*) related to the respiratory chain (GO:0070469) were dysregulated in poor-quality semen. The NADH dehydrogenase complex provides nearly 40% of the protons for ATP generation in the mitochondria (87). It helps in the transfer of electrons to the oxygen molecules of electron receptors from NADH. The energy produced during this electron transfer is deposited as ATP (88). The point mutations, copy number variations, and deletion in mitochondrial genes are associated with the diminished activity of sperm. The variations in the mitochondrial NADH dehydrogenase subunit 6 (*ND6*) gene were correlated with the total fertilization failure (89). Nucleotide variations and mutation in sperm mitochondrial NADH dehydrogenase subunit 3 (*ND3*) gene was found in asthenospermic patients (90). In the present study, we observed that both the genes (*ND3* and *ND6*) related to the respiratory chain were downregulated in the poor-quality semen indicating the unhinged mitochondrial respiratory chain complex in the sperm of poor-quality ejaculates. In poor-quality semen, we observed that four genes (*CYTB, ND6, ND4*, and *ND3*) involved in oxidative phosphorylation (bta00190) pathway were dysregulated. Among these genes, *CYTB* and *ND4* were upregulated, while ND6 and ND3 were downregulated in poor-quality semen. The transcripts like ND3, ND4, and ND6 belong to the NADH dehydrogenase complex, and the role of ND3 and ND6 was discussed earlier. A negative correlation was observed between motility and the copy number of ND4 and CYTB, in which the correlation between CYTB and motility was significant. Oxidative phosphorylation and glycolysis are the pathways involved in the production of ATP for sperm function. Oxidative phosphorylation produces ATP for sperm mitochondria, whereas glycolysis produces ATP in the head and principal piece (91). Oxidative phosphorylation is essential for the bull spermatozoa to acquire energy for capacitation (92). The dysregulation of this pathway is indicative of altered energy metabolism in poor-quality semen.

#### Perturbed Odorant Binding and Olfactory Transduction

The olfactory receptors were reported earlier in many non-olfactory tissues including testis, accessory sex glands, and sperm (93). We found that 39 genes involved in molecular function such as olfactory receptor activity (GO:0004984) and 38 genes for G-protein-coupled receptor activity (GO:0004930) were dysregulated in poor quality semen. Among these genes, *OR2T11* was the only known upregulated gene involved in both of these functions, while the remaining genes involved in these molecular functions were downregulated. Both of these molecular functions were inter-related since the mammalian olfactory receptors (OR) are the G-protein-coupled receptors located in the nasal olfactory sensory neurons and initiate the transduction of chemical stimuli into electrical signals (94). Moreover, the significant finding of this study is that the olfactory transduction is the single most highly dysregulated pathway in poor-quality semen, since 85.2% (46 genes) of the enriched dysregulated genes were involved in this pathway. In addition, the olfactory genes such as *OR2T11* and *OR10S*1 were dysregulated in both NGS sequencing and RT-qPCR in our study affirming the connection between olfactory genes and semen quality. The olfactory system can distinguish a large number of different odorant molecules, but we observed that the odorant binding (GO:0005549)-related genes were dysregulated in poor-quality semen.

Guidance mechanism of sperm in the female reproductive tract happens *via* thermotaxis, rheotaxis, and chemotaxis. The chemo-attraction occurs solely in capacitated sperm with unreacted acrosome (95). Oocyte releases chemicals to attract the capacitated sperm, but the sperm motility toward oocyte also depends on the thermal gradient and the chemicals released from the oviduct (96). The receptors on the sperm that recognizes these chemicals can smell their way to the oocyte (97). The chemicals released from the oocyte and oviduct binds with the olfactory receptors on the sperm that belong to G-protein-coupled receptors (GPCR) family, and they can increase the intracellular calcium concentration in sperm, which is essential for capacitation and acrosomal reaction, and causes sperm hyperpolarization and increased motility *via* activation of calcium-dependent chloride channels (98). This shows the link between olfactory receptors in sperm and capacitation. It was ascertained by studies in pig and giant panda showing a relationship of olfactory transduction pathway with sperm quality including cryotolerance. Furthermore, it was also reported that mRNA expression of olfactory receptors is dysregulated during cryopreservation (99, 100). These findings are in accordance with our results since we observed altered mRNA expression of olfactory receptors and pathways in poor-quality semen. The results of earlier studies and the current study clearly indicate the alterations in olfactory receptors in poor-quality semen. Further studies are required on the mRNA expression and the proteins of olfactory receptors in testis, epididymis, accessory sex glands, freshly ejaculated sperm, and cryopreserved sperm to precisely determine the role of olfactory receptors in sperm functions, semen quality, and fertility.

## Conclusion

Expression of genes involved in biological processes, such as G-protein-coupled receptor function, detection of chemical stimulus involved in sensory perception, sensory perception of smell, signal transduction, and signal synaptic transmission, was altered in spermatozoa of bulls producing poor-quality ejaculates. Both NGS and RT-qPCR analysis indicated that the olfactory transduction pathway (85% of enriched dysregulated genes were involved in this pathway) is the single most highly significantly dysregulated pathway in spermatozoa of bulls producing poor-quality semen. Since olfactory receptors and pathway are attributed to sperm chemotaxis and fertilization of oocyte, the findings of the current study assume significance and open up a new avenue for research in understanding the events associated with poor-quality semen in bulls.

## Data Availability Statement

The datasets presented in this study can be found in online repositories. The names of the repository/repositories and accession number(s) can be found at: https://www.ncbi.nlm.nih.gov/sra/?term=PRJNA772048.

## Ethics Statement

The animal study was reviewed and approved by Institute Animal Ethics Committee.

## Author Contributions

TK: contributed in the investigation, methodology, visualization, and writing of the original draft. DD: reviewed and edited the draft and was in charge of the supervision. AK: conceptualized the study and handled the project administration, resources, supervision, writing, review, and editing of the draft. MS and PN: performed the data curation and formal analysis. EK: also contributed in the methodology, writing, reviewing, and editing of the manuscript. JE: handled the methodology and investigation. TD: reviewed and edited the manuscript and was also in charge of the resources. AM: performed the formal analysis and assisted in the writing, reviewing, and editing of the manuscript. SJ and KR: contributed in the writing, reviewing, and editing of the manuscript. All authors contributed to the article and approved the submitted version.

## Funding

The present work was funded by Bill and Melinda Gates Foundation (Grant Number PP1154401) project entitled Molecular markers for improving reproduction in cattle and buffaloes.

## Conflict of Interest

The authors declare that the research was conducted in the absence of any commercial or financial relationships that could be construed as a potential conflict of interest.

## Publisher's Note

All claims expressed in this article are solely those of the authors and do not necessarily represent those of their affiliated organizations, or those of the publisher, the editors and the reviewers. Any product that may be evaluated in this article, or claim that may be made by its manufacturer, is not guaranteed or endorsed by the publisher.
