# Supplementary material for: Sperm Transcripts Associated With Odorant Binding and Olfactory Transduction Pathways Are Altered in Breeding Bulls Producing Poor-Quality Semen
Source: Front Vet Sci. 2022 Feb 22;9:799386. doi: 10.3389/fvets.2022.799386 (PMC8902071; doi:10.3389/fvets.2022.799386)
Supplement: Supplementary file 1 [file Table_1.doc]

**Supplementary table 1.** **Ejaculate Rejection Rate of Experimental bulls**

| **Bull number** | **Number of ejaculates collected** | **Number of ejaculates rejected** | **Ejaculates Rejection Rate (ERR%)** |
| --- | --- | --- | --- |
| 1 | 106 | 72 | 67.92 |
| 2 | 110 | 74 | 67.27 |
| 3 | 23 | 15 | 65.22 |
| 4 | 109 | 70 | 64.22 |
| 5 | 107 | 68 | 63.55 |
| 6 | 83 | 51 | 61.45 |
| 7 | 111 | 48 | 43.24 |
| 8 | 88 | 31 | 35.23 |
| 9 | 110 | 35 | 31.82 |
| 10 | 27 | 8 | 29.63 |
| 11 | 109 | 32 | 29.36 |
| 12 | 92 | 27 | 29.35 |
| 13 | 118 | 31 | 26.27 |
| 14 | 103 | 27 | 26.21 |
| 15 | 20 | 5 | 25.00 |
| 16 | 107 | 26 | 24.30 |
| 17 | 105 | 24 | 22.86 |
| 18 | 105 | 24 | 22.86 |
| 19 | 90 | 20 | 22.22 |
| 20 | 106 | 23 | 21.70 |
| 21 | 83 | 18 | 21.69 |
| 22 | 97 | 21 | 21.65 |
| 23 | 19 | 4 | 21.05 |
| 24 | 110 | 23 | 20.91 |
| 25 | 92 | 19 | 20.65 |
| 26 | 111 | 22 | 19.82 |
| 27 | 92 | 16 | 17.39 |
| 28 | 71 | 12 | 16.90 |
| 29 | 91 | 15 | 16.48 |
| 30 | 26 | 4 | 15.38 |
| 31 | 112 | 16 | 14.29 |
| 32 | 107 | 15 | 14.02 |
| 33 | 107 | 15 | 14.02 |
| 34 | 58 | 8 | 13.79 |
| 35 | 111 | 14 | 12.61 |
| 36 | 110 | 13 | 11.82 |
| 37 | 19 | 2 | 10.53 |
| 38 | 113 | 11 | 9.73 |
| 39 | 107 | 10 | 9.35 |
| 40 | 111 | 9 | 8.11 |
| 41 | 110 | 7 | 6.36 |
| 42 | 105 | 6 | 5.71 |
| 43 | 108 | 6 | 5.56 |
| 44 | 111 | 6 | 5.41 |
| 45 | 109 | 5 | 4.59 |
| 46 | 109 | 5 | 4.59 |
| 47 | 110 | 5 | 4.55 |
| 48 | 110 | 5 | 4.55 |
| 49 | 112 | 5 | 4.46 |
| 50 | 111 | 3 | 2.70 |


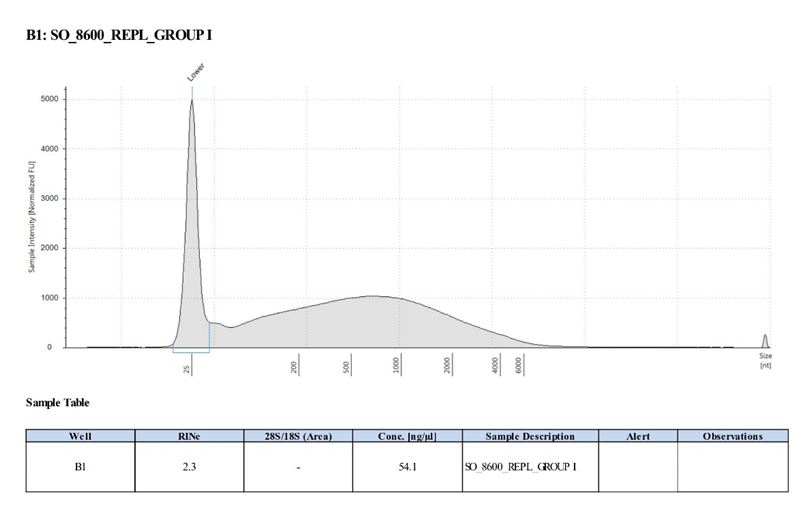


**Supplementary figure 1a: RNA integrity assessment of poor-quality bull spermatozoa**


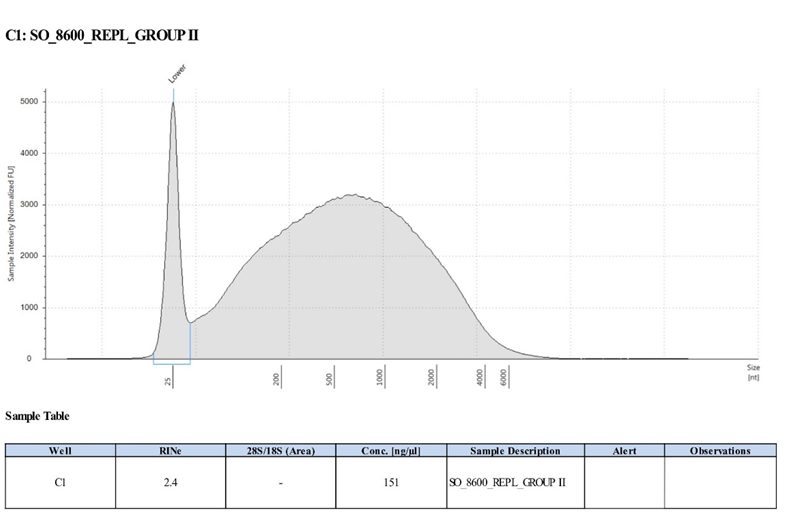


**Supplementary figure 1b: RNA integrity assessment of good-quality bull spermatozoa**


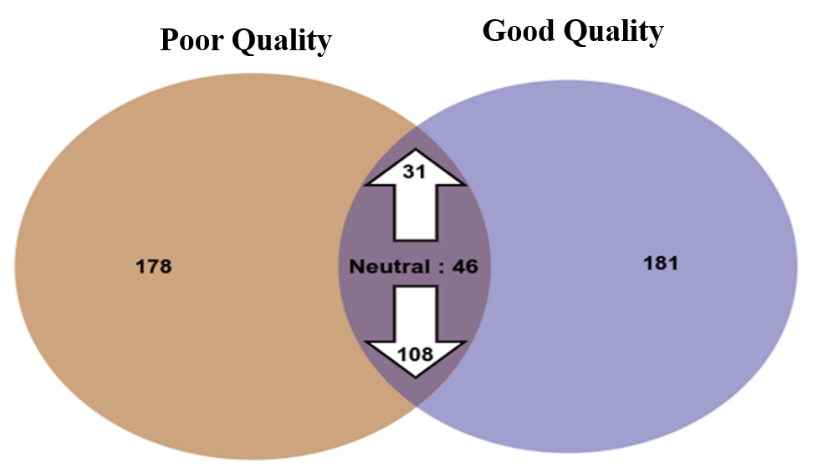


**Supplementary figure 2: Total number of transcripts and dysregulated transcripts detected in good & poor-quality bull sperm after total hit normalization of data**
